# A new method for repairing internal carotid artery rupture in skull base surgery: a case report and review of the literature

**DOI:** 10.3389/fsurg.2025.1673961

**Published:** 2025-11-27

**Authors:** Jia-Nan Li, ZhongCheng Wen, Jiajie He, Minghao Zhou, Jun Liu, Qingbo Liu, Fuyong Li, Peng Cao, Chunyong Yu, Guobiao Liang

**Affiliations:** 1 Department of Neurosurgery, General Hospital of Northern Theater Command, Shenyang, Liaoning, China; 2 Third Department of Neurosurgery, Liaoning Provincial People’s Hospital, Shenyang, Liaoning, China

**Keywords:** recurrent pituitary adenoma, skull base surgery, internal carotid artery rupture, vascular repair, complication

## Abstract

Internal carotid artery rupture during skull base surgery represents a rare yet catastrophic complication, with limited reports documented in the literature. In this article, we describe a case of ICA rupture encountered during supratentorial craniotomy for recurrent pituitary adenoma resection. The rupture was successfully managed through a novel microsurgical repair technique, achieving complete tumor resection. A 48-year-old female presented with a history of progressive visual impairment in both eyes for the past two years, 14 years after the initial pituitary adenoma resection. Reoperation was performed through the original pterional incision. During tumor dissection, sudden rupture of the right ICA occurred. Immediate cervical common carotid artery compression was applied, followed by microsurgical hemostasis using absorbable gelatin sponge and cottonoids. Temporary aneurysm clips were placed to achieve proximal flow control, allowing full exposure of the ICA rupture site. A small autologous muscle patch was used to cover the defect, and microsurgical repair was performed using a figure-of-eight suture technique. Complete hemostasis and total tumor resection were achieved. Postoperative digital subtraction angiography confirmed the patency of the ICA without stenosis, and the patient exhibited no neurological deficits postoperatively. This case combined the advantages of muscle tissue application and surgical suturing. The use of autologous muscle tissue promoted platelet aggregation, reducing tension at the rupture site and preventing further vascular tearing, while rapid suturing minimized blood loss. Additionally, the proficient collaboration and coordination with the anesthesiologist and surgical assistants were vital for controlling the intraoperative hemorrhage and ensuring prompt repair.

## Introduction

Internal carotid artery (ICA) rupture during craniotomy is a rare and catastrophic surgical complication, with an incidence of approximately 1.62%–8% (1, 2). The management principles must balance immediate hemostasis and long-term vascular patency. Current techniques are diverse and evolving; requiring careful consideration of the injury location, rupture morphology, surgical field space, and the patient's ability to compensate with collateral circulation. This places high demands on the surgeon's timely judgment.

Some scholars have conducted systematic reviews on these complications, summarizing previous vascular repair techniques and proposing an algorithm for vascular injury repair. These techniques include direct vessel suturing, the use of biomaterials, and autologous tissue repair. Each technique has its unique advantages, but the drawbacks of these methods are also evident (2) (Table 1). This article presents a case of ICA rupture and emergency intraoperative repair during a craniotomy for recurrent pituitary adenoma resection, illustrating a novel vascular repair technique. Written informed consent was obtained from the patient for publication of this case report and any accompanying images. Additionally, this article reviews and summarizes recent literature on the repair of ICA ruptures during carotid artery surgery.

**Table 1:** The advantage and disadvantage of various techniques for repairing an ICA rupture.

Techniques	Advantages	Disadvantages
Direct anastomosis	Immediate repair method with definitive repair outcome	Not applicable to complex injuries involving irregular defects
NBCA injection	Rapid hemostasis	Inflammatory vascular reactions and risk of vascular occlusion
Autologous muscle tissue	Adhesiveness, sterility, flexibility	Risk of vascular occlusion
Single-clip device	Reducing vascular occlusion time	Only applicable to endoscopic surgery
Artificial synthetic patches	Treat blood-blister-like aneurysms	Risk of vascular stenosis
Sundt clip graft	Applicable to arterial tears with irregular edges	Bulky and cumbersome design, risk of compromising perforating vessels
Venous graft and bone cement	ICA injuries within bony canals	Not applicable to other injuries
Bipolar electrocautery	Blood-blister-like aneurysms or Minor injuries	Risk of vascular stenosis
Aneurysm clip remodeling	Maintaining patency of the parent vessel	Risk of vascular stenosis
Occlusion of the ICA	Salvage option	ICA sacrifice
Revascularization technique	Reduce ischemic complications	Complex procedures and thorough preoperative preparation
Endovascular interventional technique	Maintaining patency of the parent vessel	Postoperative bleeding risk of antiplatelet therapy

## Case description

**Figure 1:**
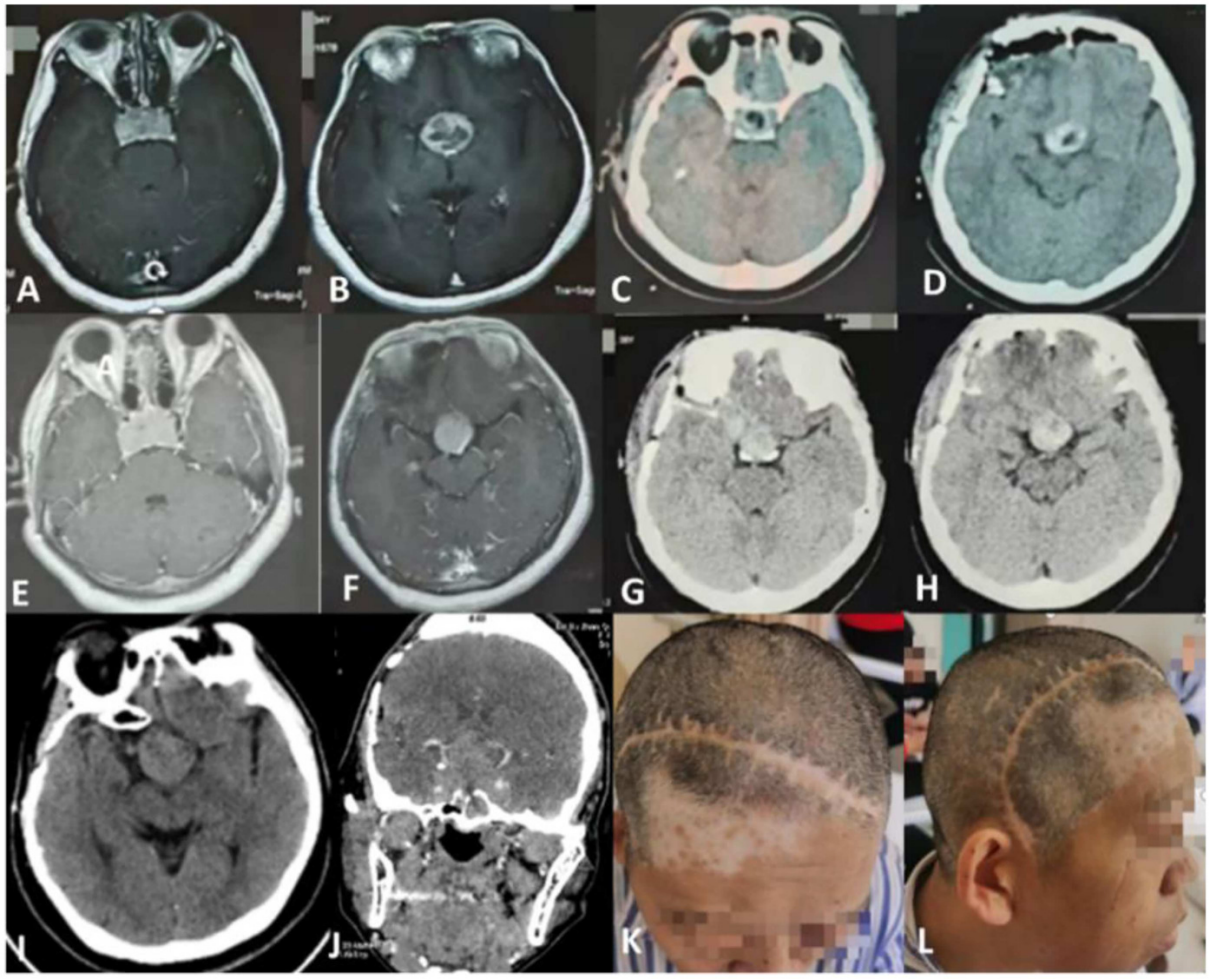
**(A,B)** preoperative imaging in another hospital in 2011; **(C,D)** postoperative imaging in another hospital in 2011; **(E,F)** preoperative imaging in another hospital in 2014; **(G,H)** postoperative imaging in another hospital in 2014; **(I,J)** preoperative imaging in our hospital; **(K,L)** original surgical incision.

A 48-year-old female presented with a history of progressive visual impairment in both eyes for the past two years, 14 years after the initial pituitary adenoma resection. The patient had previously undergone two pituitary adenoma resections via a pterional approach at another hospital, with postoperative pathology confirming a pituitary adenoma. Ophthalmologic examination revealed tubular vision in the right eye and temporal visual field defect in the left eye. Laboratory tests were within normal ranges. Endocrinological assessment demonstrated elevated growth hormone (17.894 ng/mL; normal range: 0.01–3.607 ng/mL). No significant medical or family history was noted. Contrast-enhanced CT showed a well-defined, round-shaped mass in the sellar and suprasellar regions with marked enhancement, measuring 3.7 × 3.8 × 4.4 cm. The mass displaced the Willis circle laterally, with the tumor partially encasing both internal carotid arteries, classified as Knosp grade 4 (Figure 1).

**Figure 2:**
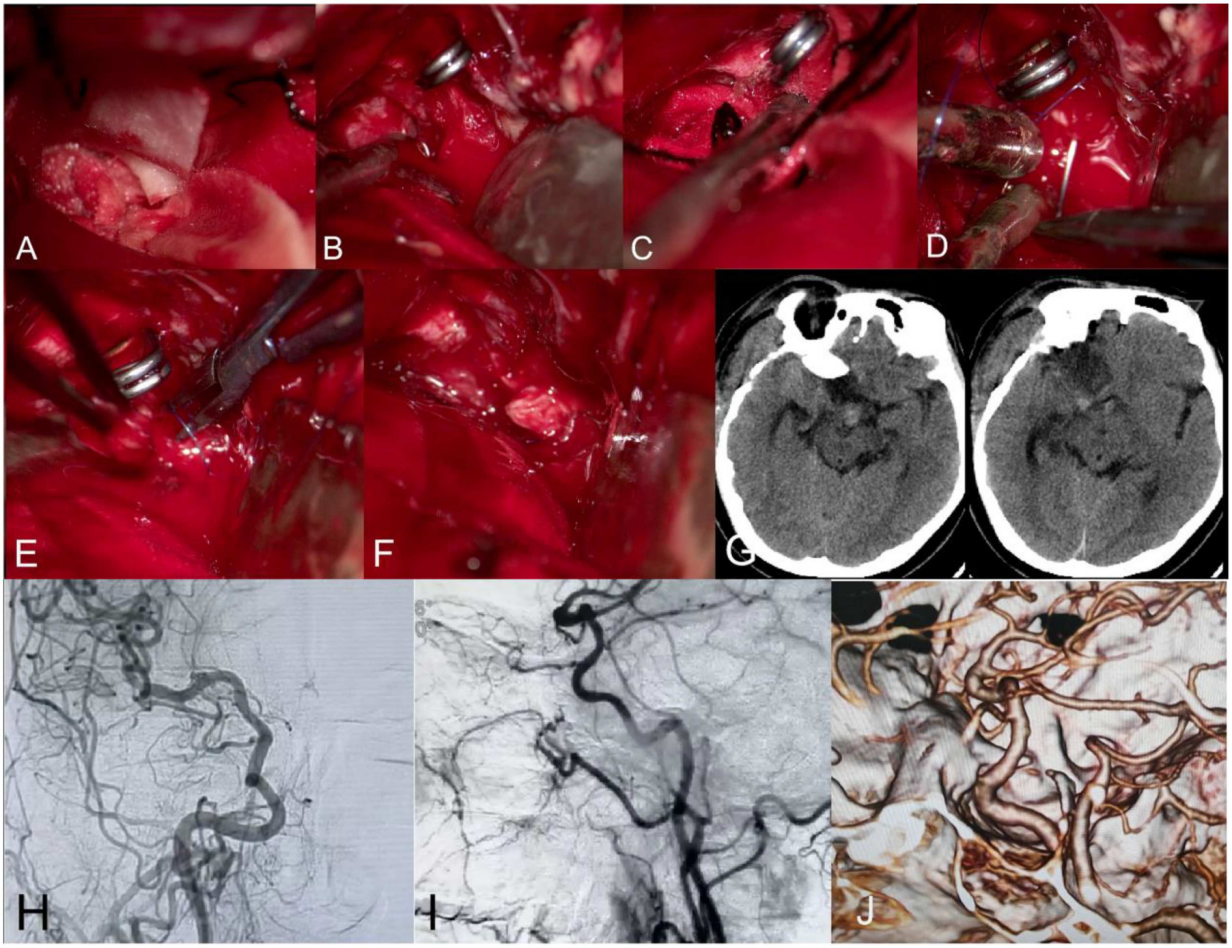
**(A)** compression hemostasis by hemostatic sponge and cotton pads; **(B)** separate and block the C6 segment of the internal carotid artery temporarily; **(C)** expose the rupture of the internal carotid artery; **(D,E)** muscle tissue is covered by the rupture of the internal carotid artery, and the rupture is repaired by micro-suturing; **(F)** temporary blockage removed and hemostasis achieved successfully; **(G–I)** postoperative CT and DSA. **(J)** 6-months follow-up CTA.

The patient was positioned supine with slight leftward head rotation. A craniotomy was performed via the previous pterional incision. During tumor dissection, torrential hemorrhage occurred from the superomedial aspect of the right ICA C7 segment. Failed conventional hemostasis precipitated immediate hemodynamic collapse, prompting the anesthesiologist to initiate manual compression at the cervical common carotid artery. Under microscopic visualization, hemostatic agents were applied while dissecting the proximal ICA (C6 segment) for temporary clip placement. After achieving proximal flow control, the arterial defect was repaired using 6-0 Prolene suture (Ethicon) with figure-of-eight technique, augmented by an autologous muscle graft interposition. The size of the autologous muscle is about 5 × 2 mm and not crushed. The suture depth was full-thickness. Complete hemostasis was verified following 20-minute temporary occlusion. Temporary elevation of blood pressure was used to maintain intracranial perfusion and cerebral protection. Intraoperative transfusion totaled 1,100 mL (800 mL packed red blood cells, 300 mL autologous blood). Gross total tumor resection was achieved with preserved neurological functions. Postoperative DSA confirmed patency of the repaired vessel (Figure 2). The patient has not experienced any new clinical neurological deficits since the surgery, and aspirin was not given after operation. Due to metal implants of other surgery, the early postoperative MRI was not performed. The 6-months follow-up CTA showed that the ICA was patency and no pseudoaneurysm.

## Discussion

ICA rupture occurs in up to 8% of skull base surgeries, yet reports on managing this complication remain scarce (3). Based on current evidence, scholars propose the following management protocol: After localizing the rupture site, blood pressure control should be considered depending on the hemorrhage volume and bleeding rate. If immediate hemostasis is achievable, select appropriate repair techniques based on defect morphology. If hemorrhage cannot be rapidly controlled, emergency ICA occlusion should be performed, followed by immediate angiography to evaluate collateral circulation and guide subsequent endovascular treatment or revascularization.

**Figure 3:**
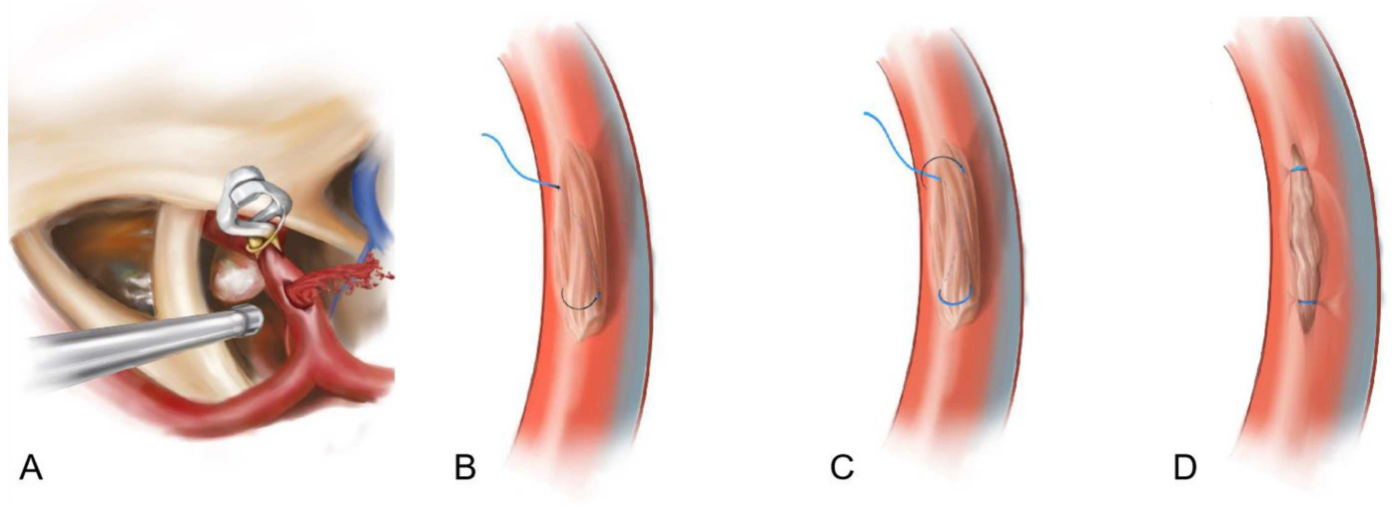
**(A–D)** pattern diagram.

Among various vascular repair techniques, suturing remains the gold standard. Direct anastomosis or suturing under temporary occlusion clip assistance demands exceptional microsurgical skills and requires thorough evaluation of collateral circulation. Sekhar et al. employed temporary occlusion techniques with 8-0 needle sutures to directly repair carotid artery rupture sites. Among their four treated cases, two patients achieved favorable outcomes, two experienced mild disability, and postoperative angiography confirmed patency in all repaired arteries. Direct suturing of vascular defects, as the most immediate repair method, provides the most definitive repair outcome. When prolonged suturing time is required, it can be combined with temporary proximal and distal occlusion of the ICA. This technique is generally suitable for ICA injuries with well-defined defects and low wall tension. For complex injuries involving irregular defects, direct suturing becomes technically challenging and may risk iatrogenic injury to the ICA (4, 5). For arterial defects under high wall tension, thorough evaluation of collateral circulation is prerequisite. This necessitates adequate mobilization of the ICA through circumferential dissection, partial vessel transection to reduce defect tension, followed by temporary occlusion and defect repair (6). In this case, the vascular defect exhibited a well-defined morphology but was under significant tension, compounded by severe local tissue adhesion from multiple prior surgeries. Direct suturing carried risks of further vascular tearing. Therefore, following temporary proximal occlusion, the “muscle coverage with internal figure-of-eight suturing technique” was employed. The figure-of-eight suturing technique, originally designed for high-tension tissue closure (e.g., muscle or aponeurosis), leverages its capacity to manage high-tension defects. Autologous muscle was utilized to buffer tension and provide secure anchoring points for traction sutures. This approach not only mitigated the risk of iatrogenic tearing inherent to direct suturing but also capitalized on the adhesive properties, sterility, and tissue compatibility of autologous muscle (Figure 3).

In addition to suturing techniques, sutureless approaches for managing carotid artery ruptures have been widely adopted with advancements in material science and interventional techniques. Requejo et al. employed α-cyanoacrylate n-butyl ester (NBCA) injection at the arterial rupture site for hemostasis. NBCA, a radiolucent permanent liquid embolic material, induces coagulation upon contact with blood and is extensively utilized in treating cerebral and spinal arteriovenous malformations and dural arteriovenous fistulas. By injecting NBCA into the ICA rupture site and the surrounding subarachnoid space, rapid hemostasis is achieved as the material solidifies and hardens upon blood contact. However, this method is not a long-term solution. Primarily, NBCA, as a chemical agent, may induce inflammatory vascular reactions through prolonged stimulation of the ICA, leading to vascular occlusion. This approach is only applicable to cases with partial vascular wall incisions and demonstrates limited efficacy for complete vascular wall lacerations (7). Van Der Veken J et al. applied autologous muscle tissue to occlude arterial ruptures, achieving hemostasis. Likely due to platelet aggregation mechanisms, autologous muscle adheres rapidly to bleeding points and effectively controls hemorrhage compared to hemostatic materials like gauze or cottonoid patties, making it suitable for managing high-flow, high-pressure arterial ruptures. Additionally, muscle offers advantages such as adhesiveness, sterility, flexibility, and easy intraoperative accessibility. However, prolonged pressure application and “overpacking” with muscle may lead to vascular occlusion, with intraoperative precision in controlling the degree of compression posing challenges (8). Romani et al., while clipping a cavernous sinus segment aneurysm of the ICA via the lateral supraorbital approach, encountered rupture of the aneurysm fundus during extradural removal of the anterior clinoid process using a rongeur. The ICA was repaired with a single-clip device, achieving good arterial repair with patent blood flow. The advantage of this method lies in shortening operative duration and reducing vascular occlusion time. This technique is currently recommended for endoscopic surgery, while its application in microsurgery requires further clinical investigation (7) 4. The method of wrapping damaged blood vessels with artificial synthetic patches [polytetrafluoroethylene [PTFE], polyester [Dacron], etc.] or autologous tissues (dura mater, blood vessels, or muscle) is widely employed (9, 10). It is commonly used to treat blood-blister-like aneurysms and for emergency management of intraoperative ICA injuries. The synthetic materials or autologous tissues are fixed in place with clips to encase the rupture site. The wrapping pressure should be sufficient to provide structural support to seal the defect, yet not excessive to avoid vascular stenosis and potential hemodynamic disturbances (11–14). Thoralf Sundt et al. developed the Sundt Clip Graft neurosurgical vascular clip to address intraoperative intracranial arterial laceration injuries and fusiform dissecting aneurysms. The inner surface of the clip is lined with Teflon, and when positioned over the vascular defect, its spring mechanism applies pressure to the synthetic graft, effectively sealing and repairing the defect (4, 7, 8). The clip is available in various sizes to accommodate intracranial arteries of different diameters: 5–6 mm clips are suitable for the ICA, while 2.5 mm clips are designed for the middle, anterior, and posterior cerebral arteries. The advantage of this technique lies in its ability to repair vascular damage while maintaining vascular patency and integrity, particularly suitable for arterial tears with irregular edges where direct suturing is challenging. However, its limitations include two aspects: Firstly, the bulky and cumbersome design may interfere with surrounding temporary occlusion clips in confined surgical fields, potentially obstructing the surgical view and complicating subsequent procedures. Secondly, vascular wall coverage might compromise perforating vessels (15, 16). Fairgrieve et al. successfully repaired an intraoperative injury of the petrous segment of the ICA using venous graft combined with bone cement (methyl methacrylate-styrene copolymer activated by methyl methacrylate) fixation. In this case report, the damaged site of the ICA was located within an inaccessible bony canal, rendering conventional repair methods nearly infeasible. By utilizing the plasticity of bone cement as a supportive overlay on the venous patch, successful repair was achieved. This method has relatively limited applicability but can be considered for ICA injuries within bony canals (17). The innovation of sutureless techniques reflects the clinical demand for rapid hemostasis. Technologies such as NBCA glue, muscle grafts, and Sundt Clip Graft vascular clips each present distinct advantages and disadvantages, requiring high case selectivity and being applicable only under specific circumstances.

Bipolar electrocautery has been utilized in the treatment of blood-blister-like aneurysms and unruptured microaneurysms (18, 19). In endoscopic surgery, it has also been successfully applied to manage iatrogenic carotid artery injuries (20). While its application in microsurgical procedures has not been reported, it may hold potential for addressing minor carotid artery breaches. The aneurysm clip remodeling technique for vascular reconstruction is theoretically feasible, but no reports have been documented regarding its application in treating iatrogenic carotid artery injuries during microsurgical skull base procedures. However, descriptions of aneurysm clip usage for ICA ruptures in endoscopic procedures have been reported (20, 21). Similar to its application in blood-blister-like aneurysm treatment, parent artery stenosis remains the most common complication (22). Occlusion of the ICA, as a salvage option, may be considered through coiling or permanent clipping to sacrifice the vessel if all aforementioned methods are unfeasible, provided that sufficient collateral circulation exists in the anterior communicating artery and/or posterior communicating artery (23–25). Revascularization techniques, being relatively complex procedures, require thorough preoperative preparation and are typically performed electively. Preoperative comprehensive evaluation of intraoperative carotid artery rupture risks should be conducted, with strict avoidance of their emergency application during surgery whenever possible (26–28). Endovascular interventional techniques, with advancements in neurointerventional and hybrid surgical procedures, theoretically represent an ideal method for repairing iatrogenic ICA injuries. Coverage of the rupture site can be achieved through deployment of covered stents, flow-diverting devices, or overlapping stent placement, thereby sealing the defect while maintaining patency of the parent vessel. A notable drawback lies in the requirement for antiplatelet therapy with stent utilization, which may elevate postoperative bleeding risks. The advent of hybrid operating rooms enables temporary flow arrest using balloon occlusion proximal to the vascular defect, thereby providing additional time for intraoperative vascular repair (29–33).

## Conclusion

Reports of carotid artery injuries during craniotomy are rare, demanding exceptional intraoperative decision-making and technical proficiency from surgeons. The internal figure-of-eight suturing technique offers advantages in managing high-tension carotid artery ruptures, while muscle tissue coverage can provide anchoring points for sutures, preventing further damage to the defect. This approach may represent an optimized repair method for intraoperative carotid artery ruptures. Further development of novel hemostatic materials or techniques is anticipated.

## Data Availability

The original contributions presented in the study are included in the article/Supplementary Material, further inquiries can be directed to the corresponding authors.

## References

[1] Muskens IS Briceno V Ouwehand TL Castlen JP Gormley WB Aglio LS The endoscopic endonasal approach is not superior to the microscopic transcranial approach for anterior skull base meningiomas-a meta-analysis. Acta Neurochir. (2018) 160(1):59–75. Eng. 10.1007/s00701-017-3390-y 29127655 PMC5735207

[2] Van Der Veken J Simons M Mulcahy MJ Wurster C Harding M Van Velthoven V. The surgical management of intraoperative intracranial internal carotid artery injury in open skull base surgery-a systematic review. Neurosurg Rev. (2022) 45(2):1263–73. Eng. 10.1007/s10143-021-01692-1 34802074

[3] AlQahtani A London NR Jr Castelnuovo P Locatelli D Stamm A Cohen-Gadol AA Assessment of factors associated with internal carotid injury in expanded endoscopic endonasal skull base surgery. JAMA Otolaryngol Head Neck Surg. (2020) 146(4):364–72. Eng. 10.1001/jamaoto.2019.4864 32105301 PMC7047871

[4] Sekhar LN Schramm VL Jr Jones NF Yonas H Horton J Latchaw RE Operative exposure and management of the petrous and upper cervical internal carotid artery. Neurosurgery. (1986) 19(6):967–82. Eng. 10.1227/00006123-198612000-00012 3808244

[5] Lussier G Evans AJ Houston I Wilsnack A Russo CM Vietor R Compact arterial monitoring device use in resuscitative endovascular balloon occlusion of the aorta (REBOA): a simple validation study in swine. Cureus. (2024) 16(10):e70789. Eng. 10.7759/cureus.70789 39493181 PMC11531354

[6] Wait SD Chang SW Killory BD White WL Spetzler RF. Anterior cerebral artery amputation and salvage repair of internal carotid artery tear: technical case report. Neurosurgery. (2009) 65(4):E820–2. discussion E822. Eng. 10.1227/01.neu.0000350978.36160.7b 19834363

[7] Requejo F Schumacher M van Velthoven V. Coating the wall of an injured intracranial carotid artery during tumor removal with n-butyl-2-cyanoacrylate: technical case report. Neurosurgery. (2006) 59(4 Suppl 2):ONSE484–5. discussion ONSE485. Eng. 10.1227/01.neu.0000232769.86686.96 17041522

[8] Van Der Veken J Mascarenhas AR Chryssidis S Poonoose SI. Management of an internal carotid artery injury during open skull base surgery with a crushed muscle patch—technical note and lessons learned. Oper Neurosurg. (2021) 21(5):356–9. Eng. 10.1093/ons/opab267 34333657

[9] Guo Y Zhang J Chen H Xu K Yu J. Suturing and dural wrapping for a blood blister-like aneurysm on the supraclinoid segment of the internal carotid artery due to dissection. World Neurosurg. (2018) 109:165–70. Eng. 10.1016/j.wneu.2017.09.186 28987850

[10] Owen CM Montemurro N Lawton MT. Blister aneurysms of the internal carotid artery: microsurgical results and management strategy. Neurosurgery. (2017) 80(2):235–47. Eng. 10.1227/neu.0000000000001259 28173470

[11] Barrow DL. Intraoperative misadventures: complication avoidance and management in aneurysm surgery. Clin Neurosurg. (2011) 58:93–109. Eng. 10.1227/neu.0b013e3182275574 21916133

[12] Feng YG Li SF Zhang PN Xin T Meng QH Tang WZ Clip-on-wrapping with dura mater to treat intracranial aneurysm neck avulsion: case reports and review of the literature. Clin Neurol Neurosurg. (2013) 115(10):2284–7. Eng. 10.1016/j.clineuro.2013.07.034 24011500

[13] Kawase T Gotoh K Toya S. A wrapping clip combined with silastic sheet for emergent hemostasis: technical note. Neurosurgery. (1994) 35(4):769–70. discussion 770-1. Eng. 10.1227/00006123-199410000-00030 7808627

[14] Kubo Y Ogasawara K Tomitsuka N Otawara Y Watanabe M Ogawa A. Wrap-clipping with polytetrafluoroethylene for ruptured blisterlike aneurysms of the internal carotid artery. Technical note. J Neurosurg. (2006) 105(5):785–7. Eng. 10.3171/jns.2006.105.5.785 17121147

[15] Park PJ Meyer FB. The sundt clip graft. Neurosurgery. (2010) 66(6 Suppl Operative):300–5. discussion 305. Eng. 10.1227/01.neu.0000369923.05148.67 20489520

[16] Lanzino G diPierro CG Laws ER Jr. Sutureless repair of major intracranial vessels with the sundt clip-graft: technical note. Acta Neurochir. (1998) 140(5):491–3. Eng. 10.1007/s007010050130 9728251

[17] Fairgrieve J Hardingham M Benjamin PJ. Repair of the intra-osseous portion of the internal carotid artery using bone cement. Br J Surg. (1981) 68(9):666–7. Eng. 10.1002/bjs.1800680919 7272697

[18] Bruneau M Amin-Hanjani S Koroknay-Pal P Bijlenga P Jahromi BR Lehto H Surgical clipping of very small unruptured intracranial aneurysms: a multicenter international study. Neurosurgery. (2016) 78(1):47–52. Eng. 10.1227/neu.0000000000000991 26317673

[19] Nussbaum ES Erickson DL. The fate of intracranial microaneurysms treated with bipolar electrocoagulation and parent vessel reinforcement. Neurosurgery. (1999) 45(5):1172–4. discussion 1174-5. Eng. 10.1097/00006123-199911000-00031 10549934

[20] Chin OY Ghosh R Fang CH Baredes S Liu JK Eloy JA. Internal carotid artery injury in endoscopic endonasal surgery: a systematic review. Laryngoscope. (2016) 126(3):582–90. Eng. 10.1002/lary.25748 26525334

[21] Fustero de Miguel D López López LB Avedillo Ruidíaz A Orduna Martínez J Casado Pellejero J Moles Herbera JA. Repair of internal carotid artery injury with aneurysm clip during endoscopic endonasal surgery: illustrative case. J Neurosurg Case Lessons. (2021) 1(6):Case2098. Eng. 10.3171/case2098 36045935 PMC9394176

[22] Bojanowski MW Weil AG McLaughlin N Chaalala C Magro E Fournier JY. Morphological aspects of blister aneurysms and nuances for surgical treatment. J Neurosurg. (2015) 123(5):1156–65. Eng. 10.3171/2014.11.jns141004 26053352

[23] Haisa T Matsumiya K Yoshimasu N Kuribayashi N. Foreign-body granuloma as a complication of wrapping and coating an intracranial aneurysm. Case report. J Neurosurg. (1990) 72(2):292–4. Eng. 10.3171/jns.1990.72.2.0292 2136243

[24] Hegde V Appaswamy S Ahluwalia H. Cranio-orbital injury with internal carotid artery laceration and a missing eyelid. Ophthal Plast Reconstr Surg. (2005) 21(6):467–9. Eng. 10.1097/01.iop.0000181348.64958.da 16304533

[25] Zhang Y Tian Z Li C Liu J Zhang Y Yang X A modified endovascular treatment protocol for iatrogenic internal carotid artery injuries following endoscopic endonasal surgery. J Neurosurg. (2020) 132(2):343–50. Eng. 10.3171/2018.8.jns181048 30684942

[26] Kalani MY Kalb S Martirosyan NL Lettieri SC Spetzler RF Porter RW Cerebral revascularization and carotid artery resection at the skull base for treatment of advanced head and neck malignancies. J Neurosurg. (2013) 118(3):637–42. Eng. 10.3171/2012.9.jns12332 23082880

[27] Klinger DR Flores BC Lewis JJ Barnett SL. The treatment of cavernous sinus meningiomas: evolution of a modern approach. Neurosurg Focus. (2013) 35(6):E8. Eng. 10.3171/2013.9.focus13345 24289133

[28] Walcott BP Lawton MT. Carotid artery occlusion and revascularization in the management of meningioma. Handb Clin Neurol. (2020) 170:209–16. Eng. 10.1016/b978-0-12-822198-3.00041-0 32586492

[29] Cobb MI Nimjee S Gonzalez LF Jang DW Zomorodi A. Direct repair of iatrogenic internal carotid artery injury during endoscopic endonasal approach surgery with temporary endovascular balloon-assisted occlusion: technical case report. Neurosurgery. (2015) 11(Suppl 3):E483–6. discussion E486-7. Eng. 10.1227/neu.0000000000000863 26284353

[30] Kocer N Kizilkilic O Albayram S Adaletli I Kantarci F Islak C. Treatment of iatrogenic internal carotid artery laceration and carotid cavernous fistula with endovascular stent-graft placement. AJNR Am J Neuroradiol. (2002) 23(3):442–6. Eng. 10.1055/s-2002-20536 11901015 PMC7975307

[31] Ghorbani M Shojaei H. Surpass streamline flow-diverter embolization device for treatment of iatrogenic and traumatic internal carotid artery injuries. AJNR Am J Neuroradiol. (2018) 39(6):1107–11. 10.3174/ajnr.A5607 29650785 PMC7410625

[32] Valentine R Wormald PJ. Carotid artery injury after endonasal surgery. Otolaryngol Clin North Am. (2011) 44(5):1059–79. Eng. 10.1016/j.otc.2011.06.009 21978896

[33] Gardner PA Snyderman CH Fernandez-Miranda JC Jankowitz BT. Management of Major vascular injury during endoscopic endonasal skull base surgery. Otolaryngol Clin North Am. (2016) 49(3):819–28. 10.1016/j.otc.2016.03.003 27267028

